# Emerging role of non-coding RNAs in glucose metabolic reprogramming and chemoresistance in colorectal cancer

**DOI:** 10.3389/fonc.2022.954329

**Published:** 2022-08-01

**Authors:** Shushan Yan, Shufeng Wang, Xinyi Wang, Wenqing Dai, Jinjin Chu, Min Cheng, Zhiliang Guo, Donghua Xu

**Affiliations:** ^1^ Department of Gastrointestinal and Anal Diseases Surgery of the Affiliated Hospital, Weifang Medical University, Weifang, China; ^2^ Medical Experimental Training Center, Weifang Medical University, Weifang, China; ^3^ Clinical Medicine of Basic Medical School, Shandong First Medical University, Jinan, China; ^4^ Central Laboratory of the First Affiliated Hospital, Weifang Medical University, Weifang, China; ^5^ Department of Physiology, Weifang Medical University, Weifang, China; ^6^ Department of Spine Surgery, The 80th Group Army Hospital of Chinese People’s Liberation Army (PLA), Weifang, China; ^7^ Department of Rheumatology of the First Affiliated Hospital, Weifang Medical University, Weifang, China

**Keywords:** Circular RNA, colorectal cancer, glucose metabolism, long noncoding RNA, metabolic reprogramming, microRNA

## Abstract

Metabolic reprogramming plays a critical role in colorectal cancer (CRC). It contributes to CRC by shaping metabolic phenotypes and causing uncontrolled proliferation of CRC cells. Glucose metabolic reprogramming is common in carcinogenesis and cancer progression. Growing evidence has implicated the modifying effects of non-coding RNAs (ncRNAs) in glucose metabolic reprogramming and chemoresistance in CRC. In this review, we have summarized currently published studies investigating the role of ncRNAs in glucose metabolic alterations and chemoresistance in CRC. Elucidating the interplay between ncRNAs and glucose metabolic reprogramming provides insight into exploring novel biomarkers for the diagnosis and prognosis prediction of CRC.

## Introduction

Colorectal cancer (CRC) is one of the most common digestive cancers, with increasing incidence and mortality in young adults in particular (1, 2). CRC is the secondly most common cancer worldwide with the incidence and mortality of 12% and 7%, respectively. Identifying novel biomarkers for the early diagnosis and treatment of CRC is very important. Targeted therapy for CRC has been promoted with progress in the high-throughput sequencing technology. Glucose metabolic reprogramming is a hallmark of cancer, which plays a critical role during carcinogenesis (3). The glucose metabolic pathways primarily include aerobic glycolysis, gluconeogenesis, and pentose phosphate pathway (PPP). Aerobic glycolysis is called Warburg effect, which is common in most aggressive cancer cells. It has been well documented that abnormal aerobic glycolysis is closely related to cancer growth and survival (4). Besides, disorders of glucose metabolic reprogramming in immune cells can lead to microenvironment imbalance and affect anti-tumor immunity (5). Research in metabolomics has suggested those key molecules underlying the metabolic mechanisms would serve as optimal approaches for CRC diagnosis and prognosis prediction. Blocking the glucose metabolic reprogramming in immune cells can help to defend against cancer.

A growing number of studies have implicated that noncoding RNAs (ncRNAs) are involved in tumor initiation and progression, primarily including circular RNA (circRNA), long noncoding RNA (lncRNA) and microRNA (miRNA) (6, 7). The ncRNAs regulatory networks are essential for tumorigenesis, tumor invasion and metastasis. Some ncRNAs act as oncogenic drivers, while some other ncRNAs function as tumor suppressors. They can regulate numerous molecular targets through RNA-RNA or RNA-protein interactions. Increasing evidence has supported that ncRNAs participate in the glucose metabolic reprogramming of cancer by targeting metabolism-associated with genes, such as glucose transporter 1 (GLUT1), hypoxia-induced factor-1a (HIF-1a), and glycogen synthase 1 (GYS1). Some ncRNAs may also influence the chemoresistance to CRC. Accumulated studies have investigated the crucial role of ncRNAs in regulating glucose metabolism and chemoresistance in CRC in the past few years (8–10). The identification of ncRNA-based glucose metabolism regulatory networks is currently emerging as a promising approach in the field of early screening and targeted therapy of CRC. In this review, we aim to elucidate the molecular targets regulated by ncRNAs that might be involved in the glucose metabolic reprogramming and chemoresistance of CRC.

## Regulation of glucose metabolic reprogramming by miRNAs

It has been well documented that abnormal glucose metabolism is one of the leading causes for CRC development (11). Usually, normal tissues acquire energy through the aerobic oxidative phosphorylation but undergo anaerobic glycolysis under hypoxia. However, the main way for acquiring energy of cancer cells is aerobic glycolysis even under the condition of adequate oxygen. Aerobic glycolysis is typical in the metabolic reprogramming of CRC. Abnormal glucose metabolism in CRC cells is primarily attributed to the mitochondrial dysfunction, abundant activation of key enzymes involved in glycolysis, altered isozyme profiles and dysregulated glucose metabolic signaling pathways (12, 13).

Accumulated studies have demonstrated that miRNAs play important roles in CRC by transcriptionally regulating specific mRNAs (14). They are single-stranded noncoding small RNAs with about 22 nucleotides. MiRNAs confer their biological effects by specifically complementary recognition of the miRNA response elements to the 3’ untranslated region (3’UTR) of mRNAs. Many miRNAs have been implicated in regulating cancer metabolic reprogramming. With advance in high-throughput technology, some miRNAs have been demonstrated to confer effects on the metabolic interactions between CRC cells and gut microbiota, suggesting the critical role of miRNAs in mediating tumor-microbiota metabolic interplays (15). To the best of our knowledge, butyrate produced by the gut microbiota provides approximately 70% of energy needs for the colonic epithelial cells. Aberrant expression of miRNAs can influence glucose metabolism mediated by butyrate in cancer cells through the gut-brain axis (16–18). Nonetheless, little is known of the potential interactions between miRNAs and gut microbiota in CRC glucose metabolic reprogramming. Current progress in the role of miRNAs in the glucose metabolism of CRC has been summarized in **Table 1**. The association between miRNAs and gut microbiota-mediated glucose metabolism in CRC has also been elucidated in the following context.

**Table 1:** MiRNAs involved in CRC glucose metabolism.

MiRNA	Expression(Up/Down)	Target	Effect	Reference
miR-4999-5p	Up	PRKAA2	Predicting CRC survival outcome; Promoting glycolysis and CRC growth	Zhang QW, et al. (19)
miR-26a	NA	PDHX	Promoting pyruvate accumulation and reducing acetyl coenzyme A production	Chen B, et al. (22)
miR-143	Down	NA	Inhibiting CRC cells proliferation and glucose uptake	Zhao J, et al. (23)
miR-149-3p	NA	PDK2	Increasing 5-FU-induced CRC cells apoptosis and inhibiting glucose metabolism	Liang Y, et al. (24)
miR-24	Up	VHL	Switching metabolism from oxidative phosphorylation to glycolysis in CRC	Jin F, et al. (20)
miR-181d	Up	CRY2 and FBXL3	Stabilizing c-myc and promoting the glucose consumption and the production of lactate in CRC	Guo X, et al. (21)
miR-488	Down	PFKFB3	Inhibiting the chemoresistance and glycolysis of CRC	Deng X, et al. (25)
miR-125b-5p	NA	NA	Inhibiting lactate generation and chemoresistance to oxaliplatin and 5-fluorouracil in CRC cells	Park GB et al. (26)
miR-339-5p	NA	hnRNPA1 and PTBP1	Inhibiting CRC cells glycolysis and growth by downregulating PKM2	Wu H, et al. (27)
miR-328	NA	GLUT1	Regulating the Warburg effect by targeting GLUT1 in CRC cells	Santasusagna S et al. (28)
miR-124, miR-137 and miR-340	NA	PKM	Switching PKM1/PKM2 ratio and regulating glycolysis rate of CRC cells	Sun Y, et al. (29)
miR-142-5p	Up	SDHB	Facilitating aerobic glycolysis of CRC cells *via* targeting SDHB	Liu S, et al. (30)
miR-374a	Down	LDHA	Refining aerobic glycolysis *via* targeting LHHA	Wang J, et al. (31)
miR-143	NA	HK2	Down-regulating HK2 and affecting glucose metabolism	Gregersen LH, et al. (32)
miR-124	Down	PRPS1 and RPIA	Inhibiting lactate production and PPP	Qiu Z, et la (33).
miR-98	Down	HK2	Functioning as a tumor suppressor and inhibiting Warburg effect	Zhu W, et al. (34)
miR-206	Down	hnRNPA1	Suppressing PKM2 expression and attenuating Warburg effect of CRC cells	Fu R, et al. (35)
miR-500a-3p	Down	CDK6	Inhibiting aerobic glycolysis and CRC progression	Liu Y, et al. (36)
miR-122	NA	PKM2	Inhibiting aerobic glycolysis in 5-FU-resistant CRC cells	He J, et al. (37)
miR-34a	NA	LDHA	Mediating inhibition of glucose metabolism in 5-FU-resistant CRC cells	Li X, et al. (38)
miR-135b	NA	SPOCK1	Promoting the Warburg effect in CRC	Babaei-Jadidi R, et al. (39)
miR-4458	Down	HK2	Refining the glycolysis and lactate Production in CRC cells	Qin Y, et al. (40)
miR-27a	Up	FBXW7	Forcing the aerobic glycolytic metabolism in CRC	Barisciano G, et al. (41)
let-7a	Up	SNAP23	Promoting EV secretion of CRCs and enhancing mitochondrial oxidative phosphorylation	Liu Y, et al. (42)
miR-101	NA	EZH2 and OGT	MiR-101-O-GlcNAc/EZH2 regulatory feedback circuit regulating CRC metabolic reprogramming	Jiang M, et al. (43)
miR-101-3p	Up	HIPK3	Promoting aerobic glycolysis by targeting HIPK3 in CRC	Tao L, et al. (44)

NA, not available.

### Glucose metabolism-associated miRNAs dysregulated in CRC

There are many glucose metabolism-associated miRNAs dysregulated in CRC, such as miR-4999-5p, miR-181d, and miR-24 (19–21) (**Table 1**). Increased expression of miR-4999-5p has been demonstrated in CRC, which can also modulate the glucose metabolic reprogramming of CRC cells by targeting the critical molecule of mTOR signaling pathway, namely, AMP-Activated Protein Kinase (PRKAA2) (19). It promotes glycolysis in CRC cells (19). MiR-26a overexpression can increase the production of pyruvate but decrease the generation of acetyl coenzyme A by suppressing pyruvate dehydrogenase protein X component (PDHX) in HCT116 cells, which suggests the critical of miR-26a in reprograming glucose metabolism in CRC (22). Jin F and the colleagues have illustrated that HIF-1α-induced miR-23a∼27a∼24 cluster promoted the progression of CRC by reprogramming glucose metabolism from oxidative phosphorylation to glycolysis (20). MiRNAs of miR-124, miR-137 and miR-340 have been documented to regulate Warburg effect by alternatively splicing pyruvate kinase isozyme (PKM) gene and controlling the ratio of PKM1/PKM2 in CRC cells (29). Another study by Qiu Z et al. have reported that miR-124 reduced PPP by regulating phosphoribosyl pyrophosphate synthetase 1 (PRPS1) and ribose-5-phosphate isomerase-A (RPIA) in CRC (33). Taken together, these findings have suggested the pivotal role of miRNAs in regulating glucose metabolism in CRC. MiRNAs exert biological effects on the glucose metabolism of CRC cells by regulating different genes. It must be mentioned that the targeted genes participating in the glucose metabolic reprogramming of CRC are diverse and complicated. The same miRNA may have different targets, while some different miRNAs may simultaneously target the same gene.

### MiRNAs and key enzymes/transporters in glycolysis

**Figure 1:**
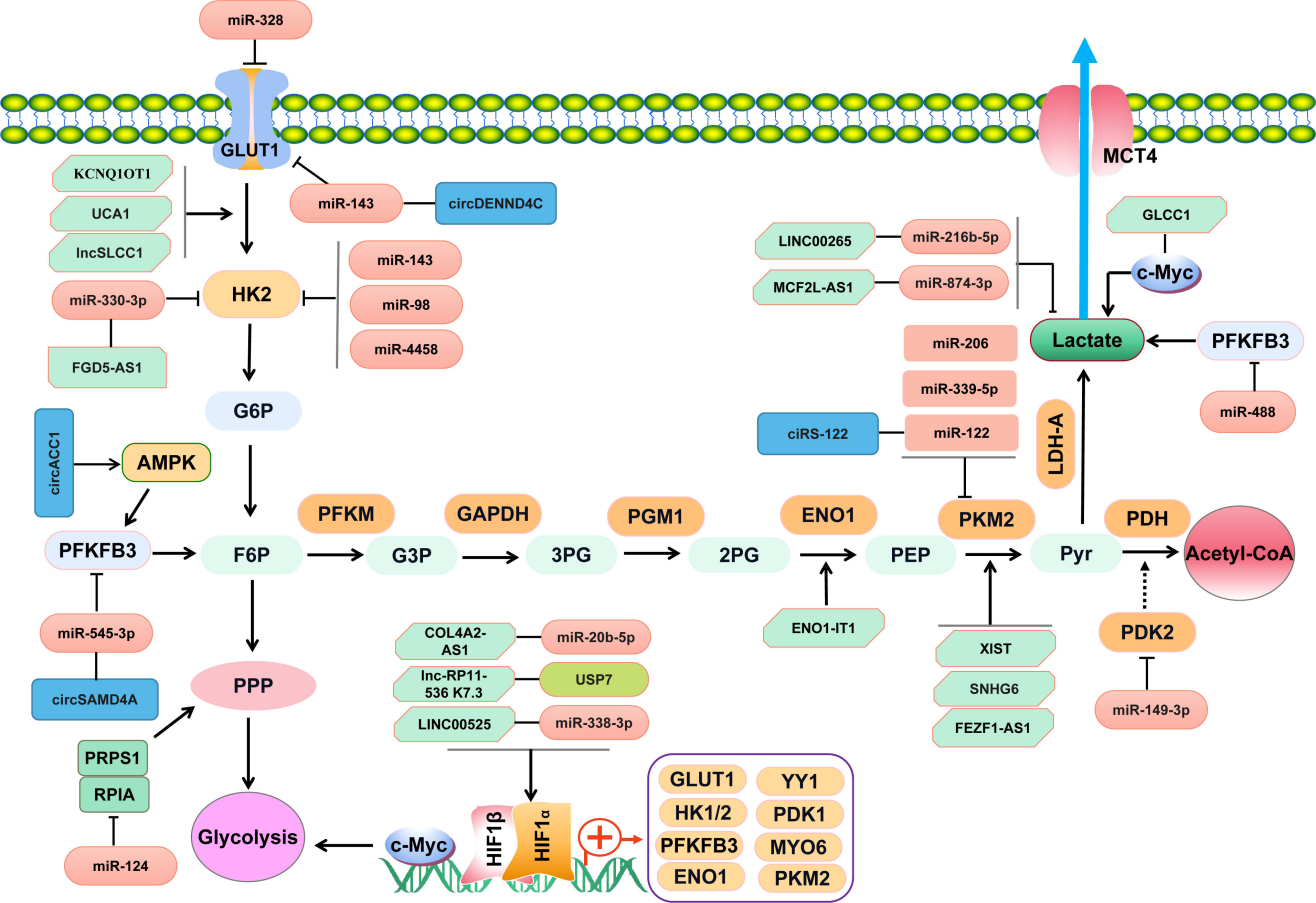
NcRNAs regulate key enzymes/transporters involved in glucose metabolism of CRC through complicated signaling pathways.

There are a couple of key enzymes and transporters regulating cancer cells glycolysis, such as GLUT1, PKM2, pyruvate dehydrogenase kinase isoform 2 (PDK2), and Lactate dehydrogenase A (LDHA). A number of miRNAs are implicated in targeting the key enzymes or transporters involved in cancer cells glycolysis (**Table 1** and **Figure 1**). Zhao J et al. have reported that miR-143 downregulated the expression of GLUT1 and inhibited glucose uptake in CRC cells (23). Besides, miR-328 is also involved in regulating the Warburg effect by targeting GLUT1 in CRC cells (28). PKM2 is another key enzyme involved in glycolysis. MiR-339-5p has been well documented to restrain CRC cells glycolysis and growth by downregulating PKM2 (27). Similarly, the study by Fu R and the colleagues have found that hnRNPA1 was a direct target of miR-206, which suppressed PKM2 expression and attenuated Warburg effect of CRC cells (35). Moreover, the upregulation of miR-142-5p inhibits the intake of oxygen but facilitates aerobic glycolysis of CRC cells by targeting the key enzyme of succinate dehydrogenase-B(SDHB) (30). Elevated glucose consumption and lactate generation is found in miR-142-5p-treated CRC cells (30). LDHA is identified as the targeted gene of miR-374a, which refines the aerobic glycolysis of CRC cells (31). Apart from the above-mentioned key enzymes and transporters in glycolysis, hexokinase 2 (HK2) is a critical rate-limiting enzyme for glycolysis. It has been demonstrated to be the direct target of miR-143, miR-4458, and miR-98 in CRC cells (32, 34). Suppression of miR-143 contributes to the shift towards aerobic glycolysis in CRC *via* targeting HK2 (32). MiR-4458 is demonstrated to prevent from glycolysis and lactate production by directly regulating HK2, which thus inhibits the progression of CRC (40). Accordingly, those aberrantly expressed miRNAs and targeted molecules involved in glycolysis would serve as helpful targets for CRC.

In the last decade, some exosomes-delivering miRNAs have been well documented to participate in regulating glucose metabolism in CRC by targeting key metabolic genes of HIF-1α and PGK1, such as exosomal miR-6869-5p, miR-8075, miR-5787, and miR-548c-5p (45). Let-7a is demonstrated to be enriched in extracellular vesicles (EVs) derived from CRC cells (42). The EVs-derived let-7a can promote the mitochondrial oxidative phosphorylation *via* downregulating SNAP23 in CRC cells (42). Tao L et al. have reported that exosomal miR-101-3p acts as an oncomiR in CRC, which promoted glycolysis and influenced metabolic homeostasis by targeting homeodomain-interacting protein kinase (HIPK3) in CRC cells (44). However, how those miRNAs being encapsulated in EVs and transferred to cancer cells remains largely unknown. More future studies are encouraged to elucidate the underlying molecular mechanism of EVs-delivering miRNAs in regulating glucose metabolic reprogramming in CRC.

### Altering effects of miRNAs on chemoresistance and glycolysis in CRC

Most interestingly, certain miRNAs are involved in regulating the chemoresistance and glucose metabolism in CRC. MiR-149-3p has been found to promote 5-Fluorouracil (5-FU)-induced CRC cells apoptosis and inhibit the glucose metabolism by targeting PDK2 (24). MiR-488 is obviously decreased in metastatic/recurrent CRC, which can also refine the chemoresistance and glycolysis of CRC by targeting a key enzyme involved in glucose metabolism, namely, phosphofructokinase-2/fructose-2,6-bisphosphatase 3 (PFKFB3) (25). The study by Park GB et al. has shown the evidence that miR-125b-5p could significantly inhibit lactate generation and the chemoresistance of CRC cells to oxaliplatin and 5-fluorouracil (26). PKM2 is a critical enzyme for glycolysis, which has been elaborated to be targeted by miR-122 involved in regulating glucose metabolism of CRC cells (37). Furthermore, miR-122 can inhibit glycolysis and serve as a useful therapeutic strategy overcoming 5-FU chemoresistance in CRC (37). Apart from miR-122, miR-34a is also implicated in regulating the sensitivity to 5-FU in CRC by refining glycolysis, suggesting the critical role of miRNAs during interactions between chemoresistance and glycolysis (38). Taken together, some miRNAs can function as therapeutic targets in patients with chemoresistant CRC, including miR-149-3p, miR-122 and miR-34a. Nevertheless, the possible regulatory mechanism in mediating chemoresistance and glucose metabolism balance in CRC needs to be investigated in the future.

## Regulation of glucose metabolic reprogramming by lncRNAs

### Glucose metabolism-associated LncRNAs dysregulated in CRC

Currently identified lncRNAs associated with glucose metabolism in CRC have been summarized in **Table 2**. As shown in **Figure 1**, some lncRNAs exert effects through RNA-protein or RNA-polypeptide interactions, while some lncRNAs regulate targeted genes *via* lncRNA-miRNA-mRNA competitive endogenous RNA (ceRNA) regulatory networks (46–50). A recent study has supported that lncRNA ZEB2-AS1 promoted glycolysis and regulated the expression of transforming growth factor β-activated kinase-binding protein 3 (TAB3) by adsorbing miR-188 (51). A LINC00265/miR-216b-5p/TRIM44 axis has been assured in promoting glycolysis and lactate production in CRC (52). LncRNA RAD51-AS1 is documented to bind with miR-29b and facilitate the expression of c-3p/NDRG2, which thus inhibits the glycolysis of CRC cells (53). Tang J et al. have found that lncRNA GLCC1 could enhance aerobic glycolysis by stabilizing transcriptional factor c-Myc and interacting with HSP90 chaperon (8). Similar effect of lncRNA LINRIS on aerobic glycolysis in CRC cells has been illustrated in the study by Wang Y et al. (54). LINRIS is identified to be upregulated in CRC tissues and plays an oncogenic role in CRC by promoting aerobic glycolysis *via* LINRIS-IGF2BP2-MYC axis (54). However, another study has suggested lncRNA MEG3 refined the aerobic glycolysis by depredating c-Myc in CRC cells (55). The transcription factor Yin Yang 1 (YY1) is demonstrated to regulate the expression of lncRNA MIR31HG, which can also forms a positive feedback *via* upregulating YY1 and sponging miR-361-3p (56). MIR31HG promotes the growth, glycolysis and lung metastasis of CRC cells (56). A recent study has suggested that transcription factor HIF-1a could increase the expression of lncRNA PTTG3P and contribute to glycolysis and M2 macrophage polarization in CRC (57). Accordingly, lncRNAs may serve as regulators for tumor-associated transcriptional factors of c-Myc and YY1, which can also facilitate the expression of certain lncRNAs *via* transcriptional activation loop.

**Table 2:** LncRNAs involved in CRC glucose metabolism.

LncRNA	Expression(Up/Down)	Targeted miRNA	Targeted mRNA	Effect	Reference
RAD51-AS1	Down	miR-29b	c-3p/NDRG2	Inhibiting cell proliferation, migration, invasion and glycolysis; promoting CRC progression *via* miR-29b/c-3p/NDRG2 signaling axis	Li C, et al. (53)
lncARSR	Up	miR-34a-5p	HK1	Sponging miR-34a-5p and promoting HK1-related aerobic glycolysis *in vitro* and *in vivo* in CRC	Li S, et al. (62)
MCF2L-AS1	Up	miR-874-3p	FOXM1	Enhancing the glucose consumption and lactate production *via* upregulating GLUT1 and LDHA	Zhang Z, et al. (63)
ZEB2-AS1	Up	miR-188	TAB3	Promoting aerobic glycolysis *via* miR-188/TAB3 axis	Li Y, et al. (51)
HCG11	Up	miR-144-3p	PDK4	Promoting glucose metabolism and 5-FU sensitization through miR-144-3p-PDK4-glucose metabolism pathway in CRC	Cui Z, et al. (64)
GLCC1	Up	NA	c-Myc	Enhancing aerobic glycolysis by stabilizing c-Myc	Tang J, et al. (8)
MIR17HG	Up	miR-138-5p	HK1	Promoting CRC liver metastasis and glycolysis through p38/Elk-1 signaling pathway	Zhao S, et al. (65)
LINC00525	Up	miR-338-3p	UBE2Q1	Promoting hypoxia-induced glycolysis by activating HIF-1α in CRC	Meng F, et al. (46)
ZFAS1	Up	NA	OLA1	Accelerating ATP hydrolysis and the Warburg effect in an m6A-dependent manner	Lu S, et al. (47)
lnc-RP11-536 K7.3	Up	NA	USP7	Promoting glycolysis, angiogenesis, and chemo-resistance *via* SOX2/USP7/HIF-1α axis in CRC	Li Q, et al. (66)
PTTG3P	Up	NA	YAP1	Facilitating glycolysis and CRC proliferation and progression *via* regulating YAP1	Zheng Y, et al. (48)
FGD5-AS1	NA	miR-330-3p	HK2	Enhancing glycolysis through the miR-330-3p-HK2 axis and promoting 5-Fu resistance in CRC	Gao S, et al. (67)
MIR31HG	Up	miR-361-3p	YY1	Promoting glycolysis and metastasis of CRC *via* MIR31HG-miR-361-3p-YY1 axis	Guo T, et al. (56)
COL4A2-AS1	Up	miR-20b-5p	HIF-1α	Promoting aerobic glycolysis of CRC cells *via* miR-20b-5p/HIF-1α axis	Yu Z, et al. (68)
UCA1	Up	NA	HK2 and LDHA	Contributing to Taxol resistance and promoting aerobic glycolysis in CRC	Shi H, et al. (59)
lncSLCC1	Up	NA	HK2	Promoting aerobic glycolysis and CRC growth	Yan T, et al. (60)
XIST	Up	miR-137	PKM2/PKM1	Elevating PKM2/PKM1 ratio and promoting 5-FU/cisplatin-resistance and glycolysis in CRC	Zheng H, et al. (69)
ENO1-IT1	NA	NA	ENO1	Promoting F. nucleatum-mediated glycolysis and oncogenesis *via* ENO1 pathway in CRC	Hong J, et al. (58)
MIAT	Up	miR-488-3p	IGF1R	Inhibiting CRC glycolysis *via* sponging miR-488-3p	Liu Y, et al. (49)
SPRY4-IT1	Up	NA	PDK1	Promoting aerobic glycolysis and CRC growth	Liu S, et al. (70)
KCNQ1OT1	Up	NA	HK2	Promoting colorectal carcinogenesis and glycolysis by targeting HK2	Chen C, et al. (61)
SNHG6	Up	NA	PKM2/PKM1	Elevating PKM2/PKM1 ratio and promoting glycolysis in CRC	Lan Z, et al. (71)
MEG3	Down	NA	c-Myc	Inhibiting glycolysis, glycolytic capacity, and lactate production in CRC cells	Zuo S, et al. (55)
HNF1A-AS1	Up	miR-124	MYO6	Enhancing glycolysis *via* miR-124/MYO6 axis in CRC	Guo X, et al. (50)
LINRIS	Up	NA	IGF2BP2	Promoting glycolysis *via* LINRIS-IGF2BP2-MYC axis in CRC	Wang Y, et al. (54)
LINC00265	Up	miR-216b-5p	TRIM44	Increasing glucose uptake, pyruvate and lactate production in CRC	Sun S, et al. (52)
MAFG-AS1	Up	miR-147b	NDUFA4	Promoting glycolysis *via* targeting miR-147b/NDUFA4	Cui S, et al. (72)
FEZF1-AS1	Up	NA	PKM2	Promoting pyruvate kinase activity and aerobic glycolysis in CRC	Bian Z, et al. (73)
HULC	NA	NA	LDHA/PKM2	Promoting aerobic glycolysis	Wang C, et al. (74)
DANCR	Up	miR-125b-5p	HK2	Enhancing aerobic glycolysis	Shi H, et al. (75)

NA, not available.

To the best of our knowledge, N6-methyladenine (m6A) modulators contribute to CRC. It has been shown that some lncRNAs can be regulated by m6A modulators. However, little is known about the mechanism of m^6^A reader in regulating glycolysis in CRC. IMP2, namely IGF2BP2, is a m^6^A reader. LncRNA ZFAS1 is found to augment the hydrolysis of adenosine triphosphate (ATP) and glycolysis by recruiting Obg-like ATPase 1 (OLA1) in CRC, which can be stabilized by IMP2 in an m^6^A-dependent manner (47). More studies are warranted to explore the precise effects of certain lncRNAs on altering m6A and glycolysis of CRC cells.

Most interestingly, the recent studly by Hong J et al. has implicated that lncRNA ENO1-IT1 was involved in promoting *Fusobacterium nucleatum* (*F. nucleatum*)-mediated glycolysis and oncogenesis *via* targeting histone modification-associated gene enolase1-intronic transcript 1 (ENO1) in CRC (58), suggesting a complicated interaction between microbiome and glycometabolic lncRNA. Targeting ENO1-IT1 may be useful for CRC patients with increased *F. nucleatum* in gut. More future studies are warranted to elucidate the potential association between gut microbiota and glucose-associated ncRNAs in regulating glucose metabolic reprogramming in CRC.

### LncRNAs and key enzymes/transporters in CRC glycolysis

LncRNA RAD51-AS1 has been shown to hamper glucose consumption and lactate production by inhibiting the key glycolysis enzyme HK2 and GLUT1 in CRC cells (53). Similar findings have been demonstrated in other studies published previously (59–61). LncRNA UCA1 is found to promote glycolysis *via* upregulating HK2 and LDHA in CRC cells (59). LncRNA HULC has been demonstrated to bind LDHA and PKM2 and thus promote aerobic glycolysis (74). Yan T, et al. (60) have reported that lncSLCC1 was upregulated in CRC and promoted glycolysis by transcriptionally activating HK2 (60). Another well documented lncRNA interacting with HK2 is lncRNA FGD5-AS1, which has been illustrated to promote glycolysis through the miR-330-3p-HK2 signaling network (67). Moreover, a lncRNA DANCR-miR-125b-5p-HK2 axis has been well established in colon cancer cells, which can promote aerobic glycolysis (75). Additionally, MCF2L-AS1 is found to be enriched in tissues of CRC, which enhances the glycolysis of CRC cells *via* MCF2L-AS1/miR-874-3p/FOXM1 ceRNA axis and upregulates GLUT1 and LDHA (63). It has been elaborated that lncARSR can sponge miR-34a-5p and promote hexokinase 1(HK1)-mediated glycolysis in CRC (62). Besides, high level of lncARSR predicts poor survival of CRC (62). Similarly, the study by Zhao S, et al. has shown the evidence that lncRNA MIR17HG facilitated HK1 expression by acting as a ceRNA for miR-138-5p (65). MIR17HG promotes glycolysis and the liver metastasis of CRC (65). Furthermore, LncRNA SPRY4-IT1 has been demonstrated to enhance CRC cell growth and glycolysis by promoting phosphoinositide-dependent kinase 1 (PDK1) expression (70). Similar altering effect of lncRNA MAFG-AS1 has been illustrated to promote PDK1 expression in CRC (72). Taken together, lncRNAs participate in CRC glycolysis primarily by regulating the key glycolysis-associated enzymes of HK1, HK2, PDK1, and crucial transporter GLUT1 (**Figure 1**). Elucidating the underlying mechanism and targets of lncRNAs in regulating glycolysis is helpful for exploring more effective strategies for the diagnosis and treatment of CRC.

### HIF-1a

HIF-1a is a key transcriptional factor for hypoxia-induced glycolysis in cancer, which is differentially regulated by diverse lncRNAs. LINC00525 is documented to activate HIF-1α, increase the expression of ubiquitin-conjugating enzyme E2Q family member 1 (UBE2Q1), and enhance hypoxia-enhanced glycolysis through miR-338-3p/UBE2Q1/β-catenin axis in CRC (46). Similarly, lnc-RP11-536 K7.3 plays an oncogenic role in CRC by promoting the angiogenesis, glycolysis, and chemo-resistance in CRC through the SOX2/USP7/HIF-1α signaling pathway (66). The study by Yu Z et al. has reported that miR-20b-5p was bound with lncRNA COL4A2-AS1, which facilitated the glycolysis of CRC cells by activating HIF-1α (68). Accordingly, HIF-1a-dependent lnRNAs serve as promising approaches for CRC treatment by controlling glucose metabolic balance.

### PKM2

As a key enzyme in glycolysis, PKM2 has been demonstrated to be regulated by several lncRNAs. For instance, lncRNA XIST facilitates glycolysis of CRC cells by upregulating PKM2 through XIST/miR-137/PKM2 axis (69). Similarly, lncRNA SNHG6 plays a critical role in the glucose metabolism of CRC, which specifically splices PKM mRNA, increases PKM2/PKM1 ratio and promotes the glycolysis in CRC (71). FEZF1-AS1 has been shown to promote the pyruvate kinase activity and aerobic glycolysis by targeting PKM2 in CRC cells (73). Taken together, lncRNA-PKM2 axis is critical in regulating CRC glycolysis.

### Involvement of LncRNAs in regulating CRC chemoresistance and glycolysis

Accumulating studies have suggested the critical role of lncRNAs in chemoresistance in cancer. Some lncRNAs have also been implicated to regulate the glucose metabolism and chemoresistance in CRC. The study by Li Q et al. have identified an oncogenic gene lnc-RP11-536 K7.3, which enhanced the glycolysis and chemoresistance to oxaliplatin in CRC *via* SOX2/USP7/HIF-1α signaling axis (66). Pyruvate dehydrogenase kinase 4 (PDK4) is a crucial enzyme for glucose metabolism. LncRNA HCG11 has been validated to facilitate 5-FU resistance by sponging miR-144-3p and upregulating PDK4 in CRC (64). Similar to HCG11, lncRNA FGD5-AS1 promotes glycolysis and 5-FU resistance of CRC cells by acting as a ceRNA for miR-330-3p (67). Besides, lncRNA UCA1 is documented to contribute to paclitaxel (Taxol)-resistance and promote glycolysis by facilitating the expression of HK2 and LDHA in CRC (59). Moreover, lncRNA XIST is demonstrated to promoting 5-FU/cisplatin-resistance and glycolysis in CRC by increasing the ratio of PKM2/PKM1, while miR-137 mimics can alleviate the facilitating effect of XIST (69). Accordingly, lncRNAs involved in regulating CRC glycolysis and chemoresistance will serve as novel anticancer strategies for CRC in the future.

## Regulation of glucose metabolic reprogramming by circRNAs

### Dysregulated circRNAs Associated with glucose metabolic reprogramming in CRC

**Table 3** has shown the aberrantly expressed circRNAs in CRC. Some circRNAs function as onco-circRNAs, while some others act as cancer-suppressors. CircTADA2A has been reported to inhibit the cell cycle, glycolysis of CRCs but significantly promote CRC cells apoptosis (76). CircNOX4 has been identified as an oncogenic circRNA in CRC by enhancing the glycolysis and controlling the expression of CDC28 protein kinase regulatory subunit 1B(CKS1B) in CRC cells through the circNOX4/miR-485-5p/CSK1B axis (77). Knockdown of circ_0000231 can inhibit glycolysis and the growth of CRC cells by sponging miR-502-5p, which binds to myosin VI (MYO6) (78). A recent published study has suggested that circPLCE1 could also function as a ceRNA binding with miR-485-5p and expedite epithelial mesenchymal transformation (EMT) and glycolysis of CRC cells (79). Besides, circPLCE1 is capable of promoting tumor-associated macrophage (TAM) polarization towards M2 *via* upregulating γ-Actin Gene (ACTG1) but inhibiting miR-485-5p in CRC (79). Furthermore, silencing of circ-RNF121 represses the growth and glycolysis of CRC cells, which can act as a sponge for miR-1224-5p and target forkhead box M1 (FOXM1) (80). Most importantly, circ-RNF121 can be packaged into exosomes and thus contributes to intercellular communications and regulates glycolysis in CRC (80). Apart from circ-RNF121, exosomes-delivering ciRS-122 is involved in promoting the glycolysis of CRC cells through ciRS-122/miR-122/PKM2 ceRNA network (81). Accordingly, circRNA is capable of acting as a sponge of specific miRNA and thus participates in the Warburg effect by regulating glycolysis-associated genes in CRC (**Figure 1**). Some circRNAs can be delivered by exosomes and mediate glucose metabolic reprogramming in CRC, including circ-RNF121 and ciRS-122 (**Figure 1**).

**Table 3:** CircRNAs involved in CRC glucose metabolism.

CircRNA	Expression(Up/Down)	Targeted miRNA	Targeted mRNA	Effect	Reference
circDENND4C	Up	miR-760	GLUT1	Promoting the proliferation, migration, and glycolysis of CRC cells	Zhang Z, et al. (82)
circTADA2A	Down	miR-374a-3p	KLF14	Inhibiting cell cycle, glycolysis and promoting the CRC cells apoptosis	Zheng L, et al. (76)
circNOX4	Up	miR-485-5p	CKS1B	Serving as an oncogenic circRNA and promoting the glycolysis of CRC cells through miR-485-5p/CKS1B signaling	Wang X, et al. (77)
circ_0000231	Up	miR-502-5p	MYO6	Promoting glycolysis and MYO6 expression through sponging miR-502-5p	Liu Y, et al. (78)
circPLCE1	Up	miR-485-5p	ACTG1	Promoting epithelial mesenchymal Transformation, M2 polarization and glycolysis in CRC	Yi B, et al. (79)
circSAMD4A	Up	miR-545-3p	PFKFB3	Contributing to 5-Fu resistance and promoting glycolysis	Gao Y, et al. (83)
circ-RNF121	Up	miR-1224-5p	FOXM1	Promoting CRC growth and glycolysis	Jiang Z, et al. (80)
ciRS-122	NA	miR‐122	PKM2	Encapsulating in exosomes and promoting the glycolysis and chemoresistance of CRC cells	Wang X, et al. (81)
circACC1	Up	NA	AMPK	Promoting the glycolysis and fatty acid β-oxidation	Li Q, et al. (84)

NA, not available.

### CircRNAs and key enzymes/transporters in glycolysis

The study by Zhang Z et al. has demonstrated that circDENND4C was upregulated in CRC, which promoted the proliferation, migration, and glycolysis of CRC cells by acting as a ceRNA for miR-760 and regulating GLUT1 (82). Besides, 6-phosphofructo-2-kinase/fructose-2,6-bisphosphatase isotype 3 (PFKFB3) is a pivotal enzyme for glucose metabolism. Gao Y and the colleagues have found circSAMD4A could facilitate the expression of PFKFB3 and promote glycolysis by sponging miR-545-3p (83). Moreover, PKM2 is another crucial enzyme for glycolysis in cancer. Exosomes from oxaliplatin-resistant CRC cells can deliver ciRS-122 to oxaliplatin-sensitive cells, which thereby upregulates PKM2 expression and promotes the glycolysis and drug resistance of CRC cells (81). Li Q et al. have reported that circACC1 played a critical role during the metabolic reprogramming of CRC cells by regulating adenosine monophosphate-activated protein kinase (AMPK) (84). CircACC1 enhances both fatty acid β-oxidation and glycolysis in CRC cells by activating AMPK (84). Taken together, those key enzymes or transporters targetedly regulated by circRNAs have suggested novel markers for CRC diagnosis and treatment by controlling glucose metabolism.

### Effects of circRNAs on chemoresistance in CRC by regulating glycolysis

Some dysregulated circRNAs participate in regulating chemoresistance and glycolysis in CRC, such as circSAMD4A (83) and ciRS-122 (81). CircSAMD4A contributes to 5-Fu resistance *via* targeting miR-545-3p/PFKFB3 and regulating glycolysis of CRC cells, while knockdown of circSAMD4A improves the sensitivity of 5-Fu (83). Exosomes-derived ciRS-122 is capable of promoting glycolysis and making chemosensitive-CRC cells transform into chemoresistant-CRC cells *via* miR-122/PKM2 axis (81). Accumulating studies have implicated the important role of circRNA in regulating immune metabolic reprogramming and immune microenvironment balance in carcinogenesis (85). CircPLCE1 has been documented to promote TAM polarization towards M2 through miR-485-5p/ACTG1 axis in CRC, which plays a critical role in regulating CRC immune microenvironment balance (79). All these findings have provided a promising circRNA-targeted therapy for CRC by shaping cancer glucose metabolism, immune microenvironment balance and cancer cells chemoresistance.

## Concluding remarks and future directions

In conclusion, ncRNA-based glucose metabolic reprogramming and chemoresistance have provided promising prospects for CRC. Elucidating the interaction and possible mechanism between ncRNAs and metabolic reprogramming has shed some insights into understanding the pathogenesis and drug resistance mechanisms of CRC. Novel biomarkers for the diagnosis, chemoresistance intervention and prognosis prediction of CRC can be investigated in more future studies. Most importantly, it is urgent to search for sufficient evidence supporting the practical clinical applications of ncRNAs in CRC.

## Author contributions

SY, DX and ZG designed the manuscript. SW, WD, JC, SY and XW performed literature research and drafted the article. MC, DX and ZG revised the review. All authors have read and approved the final article. All authors contributed to the article and approved the submitted version.

## Funding

This study is funded by National Natural Science Foundation, China (82003042, 82171790, and 81870237), and Natural Science Foundation of Shandong Province, China (ZR2020KC001).

## Conflict of interest 

The authors declare that the research was conducted in the absence of any commercial or financial relationships that could be construed as a potential conflict of interest.

## Publisher’s note

All claims expressed in this article are solely those of the authors and do not necessarily represent those of their affiliated organizations, or those of the publisher, the editors and the reviewers. Any product that may be evaluated in this article, or claim that may be made by its manufacturer, is not guaranteed or endorsed by the publisher.

## Abbreviations

CRC, colorectal cancer; ncRNAs, non-coding RNAs; PPP, pentose phosphate pathway; circRNA, circular RNA; lncRNA, long noncoding RNA; miRNA, microRNA; GLUT1, glucose transporter 1; HIF-1a, hypoxia-induced factor-1a; GYS1,glycogen synthase; PDHX, pyruvate dehydrogenase protein X component; 3’UTR,3’ untranslated region; PKM, pyruvate kinase isozyme; HK1, hexokinase 1; HK2, hexokinase 2; SDHB, succinate dehydrogenase-B; PRKAA2, AMP-Activated Protein Kinase; GLUT1, glucose transporter 1; PDK2, pyruvate dehydrogenase kinase isoform 2; LDHA, Lactate dehydrogenase A; EVs, extracellular vesicles; HIPK3, homeodomain-interacting protein kinase; ceRNA, competitive endogenous RNA; TAB3, transforming growth factor β-activated kinase-binding protein 3; YY1, Yin Yang 1; UBE2Q1, ubiquitin-conjugating enzyme E2Q family member 1; ATP, adenosine triphosphate; OLA1, glycolysis by recruiting Obg-like ATPase 1; AMPK, adenosine monophosphate-activated protein kinase; TAM, tumor-associated macrophage; ACTG1, γ-Actin Gene; FOXM1, forkhead box M1.
